# A Highly Efficient RF-DC Converter for Energy Harvesting Applications Using a Threshold Voltage Cancellation Scheme

**DOI:** 10.3390/s22072659

**Published:** 2022-03-30

**Authors:** Muhammad Basim, Danial Khan, Qurat Ul Ain, Khuram Shehzad, Syed Adil Ali Shah, Byeong-Gi Jang, Young-Gun Pu, Joon-Mo Yoo, Joon-Tae Kim, Kang-Yoon Lee

**Affiliations:** 1 Department of Electrical and Computer Engineering, Sungkyunkwan University, Suwon 16419, Korea; basim@skku.edu (M.B.); danialkhan@skku.edu (D.K.); quratulain@skku.edu (Q.U.A.); adilshah@skku.edu (S.A.A.S.); seeys17@skku.edu (B.-G.J.); hara1015@skku.edu (Y.-G.P.); 2 SKAiChips, Sungkyunkwan University, Suwon 16419, Korea; khuram1698@skku.edu (K.S.); fiance2@g.skku.edu (J.-M.Y.); 3 Department of Electrical and Electronic Engineering, Konkuk University, Seoul 05029, Korea; jtkim@konkuk.ac.kr

**Keywords:** self-threshold voltage cancellation (STVC), RF energy harvesting, power conversion efficiency (*PCE*), CMOS technology, RF-DC converter

## Abstract

In this paper, a self-threshold voltage (*V_th_*) compensated Radio Frequency to Direct Current (RF-DC) converter operating at 900 MHz and 2.4 GHz is proposed for RF energy harvesting applications. The threshold voltage of the rectifying devices is compensated by the bias voltage generated by the auxiliary transistors and output DC voltage. The auxiliary transistors compensate the threshold voltage (*V_th_*) of the PMOS rectifying device while the threshold voltage (*V_th_*) of the NMOS rectifying device is compensated by the output DC voltage. The proposed RF-DC converter was implemented in 180 nm Complementary Metal-Oxide Semiconductor (CMOS) technology. The experimental results show that the proposed design achieves better performance at both 900 MHz and 2.4 GHz frequencies in terms of *PCE*, output voltage, sensitivity, and effective area. The peak power conversion efficiency (*PCE*) of 38.5% at −12 dBm across a 1 MΩ load for 900 MHz frequency was achieved. Similarly, for 2.4 GHz frequency, the proposed circuit achieves a peak *PCE* of 26.5% at −6 dBm across a 1 MΩ load. The proposed RF-DC converter circuit shows a sensitivity of −20 dBm across a 1 MΩ load and produces a 1 V output DC voltage.

## 1. Introduction

In the past decade, the interest in energy harvesting for portable and wearable electronic devices, biomedical implanted devices, radio and frequency identification (RFID), and the Internet of Things (IoT) is increasing day by day [1,2,3,4,5]. However, in IoT, the near-field technique cannot scale well where wireless sensor nodes obtain power over wide indoor and outdoor environments [6]. Solar energy, RF energy, thermal energy, and vibration energy are some of the main sources for energy harvesting applications. The density of wireless devices rapidly increased in this decade. Moreover, mostly ultrahigh-frequency ISM bands are used for communication systems and harvest multiband RF energy simultaneously [7].

The concept of using RF signals as a source of power for wireless devices is quite appealing. The power associated with communication signals is unpredictable and often minimal; thus, it is difficult to use it for providing power supply to wireless electronic devices. To reduce the cost, it is necessary to integrate an RF energy harvesting system with a low-power system on a CMOS integrated chip. A far-field RF energy harvesting system is used to harvest energy from ambient RF energy sources, i.e., frequency modulation (FM) or dedicated RF sources and amplitude modulation (AM) radio transmission, cellular transmission signals, and television signals. A limited RF energy power source is transmitted for RF energy harvesting as specified by the Federal Communication Commission. This RF signal is strongly affected by a number of factors that degrade the performance of the signal, including weak signal strength and path loss. The available power source for collecting RF energy depends upon the path loss of the available RF signal in free space, which can be calculated using the following equation:

(1) $$L_{P}=10\;\log_{10}{\left(\frac{4\Pi D}{\lambda }\right)}^{2}$$

where *L_P_* is the path loss of the free space, *D* is the distance from the source, and *λ* is the wavelength of the signal. The equation shows that the free space path loss increases if the wavelength (*λ*) increases.

**Figure 1:**
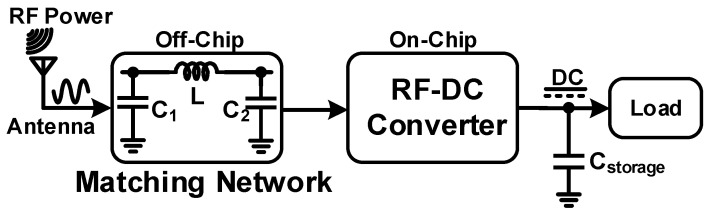
Block diagram of an RF energy harvester.

Figure 1 shows the block diagram of the proposed RF-DC architecture in which the RF-DC converter is a key component of the RF energy harvesting system that converts the incoming RF signal to DC voltage. The RF energy harvesting unit consists of an antenna, impedance matching network, radiofrequency to direct current (RF-DC) circuit, and a storage device. The antenna collects electromagnetic wave signals from the environment. The strength of the electromagnetic signal is very low and decreases rapidly as the distance from the antenna to the RF source increases. The input impedance of the RF-DC converter to a 50 Ω antenna is matched by the matching network. Moreover, it also maximizes the power transfer between them. Impedance mismatch may occur due to the varying input received from RF signals that reduce the power of the rectifier. For this purpose, a Pi-matching network is used in the proposed structure. Finally, the storage device stores the converted output DC voltage and provides for further use in wireless electronic devices. The performance of the system can be assessed based on the power conversion efficiency (*PCE*) of the RF-DC converter.

Several RF-DC converter architectures have been published which have enhanced the *PCE* for energy harvesting applications. RF energy harvesting can be adopted to operate IoT devices. RF energy harvesting is beneficial for distance flexibility. Since its output is unpredictable and small, it degrades the system’s strength. A CMOS RF rectifier proposed in [8] is based on a self-threshold voltage cancellation scheme. In the circuitry, positive feedback is used to minimize the threshold voltage of the transistors, but the disadvantage of this mechanism is that *PCE* rapidly rises and falls in response to the input RF signal. Moreover, with an increase in input RF voltage level, the RF-DC converter’s maximum output voltage saturates. A digital control loop and an on-chip capacitor array are implemented with an adaptive matching function [9]. The effect of the matching network must be considered to improve the power transfer to the rectifier. In the case of perfect matching, a pi-type matching network is used to provide a passive voltage enhancing factor. Higher efficiency can be attained to minimize the threshold voltage of MOSFETs in RF-DC circuitry. Threshold voltage causes loss in the RF-DC converter circuit, which is one of the major difficulties in design [10]. A control circuit and two differential sub-rectifiers are used in [11] for adaptive power harvesters. The rectifier is a switch between the serial and parallel modes by a control signal generated by the control circuit. The control circuit senses the output voltage of two sub-rectifiers. In reference [12], a class-E amplifier that is based on time-reversal duality theory is used as a class-E rectifier. The other methods use a voltage multiplier which makes them different from other RF rectifiers. A full-wave matching network and cross dipole antenna are proposed in [13] for a rectifier-booster regulator. This rectifier converts the RF signal to DC voltage and boosts it. A cross-connected differential rectifier is presented in [14,15] with a differential custom antenna. In [16,17], an optimum number of the rectifier is used to maintain high efficiency by using the maximum power point tracking (MPPT) technique over a wide input power range. The author in [18] reported a dual path adaptive control CMOS differential rectifier. The adaptive control circuit switches the rectifier between low- and high-power paths according to the input power level. The limitation in this research is more power loss due to the use of multiple stages of a cross-coupled rectifier. A 17-stage rectifier is presented in [19], which can deliver 1.2 V at 1 MΩ load with a self-compensated structure. A self-compensation scheme is presented in [20] in which an individual body biasing is provided to triple-well NMOS transistors. The triple-well NMOS transistors are used as a rectifying device, although triple-well NMOS transistors are not available in all CMOS technologies [21,22]. The design in [23] presents a differential cross-connected CMOS rectifier that minimizes the leakage current and compensates for the threshold voltage of the rectifying device. The design in [24,25] also proposed a self-threshold voltage cancellation scheme for RF energy harvesting applications. The referred papers also uses the threshold voltage cancellation scheme-based rectifier, which works for a low frequency of 402 MHz by using N-numbers of stages of rectifier with PMOS transistors. In [26], the author proposed a 10-stage cross-connected rectifier by using the threshold voltage compensation technique for the heavy load of 5 MΩ. A 900 MHz RF-DC converter is proposed in [27] with the aim to optimize the sensitivity and generate 50 µW output power. A multi-path energy harvesting architecture is proposed to maintain the high *PCE* of the system [28]. Similarly, in reference [29], the design uses a passive multi-stage RF to DC rectifier that is based on n-well technology. Moreover, the backward connection is used to generate the negative source bias in the Dickson charge multiplier.

The design of a highly efficient RF-DC converter using the threshold voltage cancellation scheme for energy harvesting applications is presented in this paper. This paper is organized as follows. Section 2 discusses the self-threshold voltage cancellation scheme. The proposed RF-DC converter is explained in Section 3. Section 4 presents the measurement results. Finally, Section 5 explains the conclusion of the paper.

## 2. Self *V_th_* Cancellation Scheme

**Figure 2:**
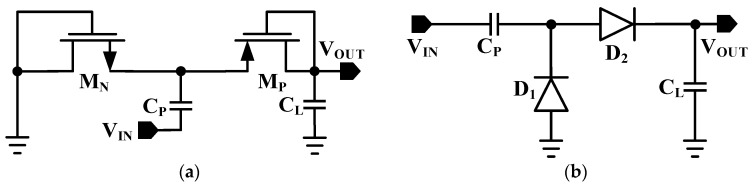
Conventional rectifier design. (**a**) CMOS-based voltage doubler. (**b**) Diode connected voltage doubler.

The *V_th_* of the rectifying device plays a significant role in the performance and operation of the RF-DC converter for energy harvesting. For an RF-DC converter operation to rectify a low RF power to DC power, a low-threshold voltage rectifying device is required. Different technology-based approaches are proposed to reduce the threshold voltage of the devices. Some of the devices include SMS, HSMS, Schottky diodes, SOS, and floating gate transistors that usually lower the threshold voltage by storing the pre-charged voltage at the gate. The additional fabrication steps are the main drawback of the technology-based approach. Moreover, it also prevents the integration of RF energy harvesters in normal CMOS ICs. To reduce the threshold voltage, an active/passive circuit technique can also be used. The active technique uses an external power source that increases the cost and maintenance, while additional circuitry is needed to compensate for the threshold voltage in the passive technique. Most of the RF-DC converter designs focused on the reduction in threshold voltage while neglecting the increase in the reverse leakage current and power losses. Consequently, decreasing the threshold voltage may cause an increase in leakage current which has an adverse effect on the output DC voltage and *PCE* of an RF-DC converter. Considering the threshold voltage and reverse leakage, the overall *PCE* can be defined as:

(2) $$PCE=\frac{P_{OUT}-P_{leakage}}{P_{IN}}$$

where *P_OUT_* is the power delivered to the load when the transistors act as forward-biased, *P_leakage_* is the output leakage power when the transistors act as reverse-biased, while *P_IN_* is the input power. The *PCE* of the proposed rectifier starts to increase if the input power is increased. To attain the highest *PCE*, several strategies for lowering the voltage drop across MOS transistors have been proposed. However, when the voltage drop across transistors is reduced, the reverse leakage current in the negative half cycle is increased. This behavior produces a decrease in the *PCE* because it introduces the energy loss stored in previous cycles. The ratio of the useable DC power given to the load resistor divided by the total input RF power delivered to the RF-DC converter is the actual *PCE* of the proposed rectifier. This *PCE* can be computed by the following equation:

(3) $$\eta \left(\%\right)=\frac{P_{OUT}}{P_{IN}}=\frac{V^{2}{}_{OUT}}{R_{L}\times P_{IN}}\times 100$$

where *V_OUT_* is the output DC voltage across the load resistor *R_L_* and *P_IN_* is the input RF power from the ambient sources. Figure 2a shows the voltage doubler based on CMOS transistors by connecting the gate and drain terminal with each other. The PMOS transistor works in a one-half cycle while the NMOS transistor works in the other half cycle of the input. Figure 2b shows the diode-based voltage doubler which converts the AC input to DC output. If the voltage drop across each transistor reaches zero, the output voltage of the voltage doubler can be double that of the amplitude of the RF signal. Therefore, the main challenge is to minimize the voltage drop across the forward-biased transistors to maximize the power flow to the output and to minimize the reverse leakage current to avoid the loss of energy.

## 3. Proposed RF-DC Converter

**Figure 3:**
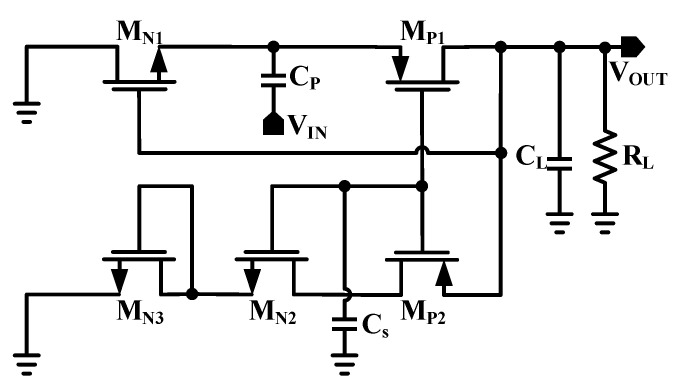
Circuit diagram of proposed RF-DC converter.

Figure 3 shows the circuit architecture of the proposed RF-DC converter. The proposed RF-DC converter uses both positive and negative levels of incoming RF signal. The rectifier cancels the threshold voltage effect using the input RF voltage and the output DC voltage. The basic RF-DC converter is designed by using a PMOS transistor (M_P1_) and an NMOS transistor (M_N1_). The threshold voltage compensation circuitry in the proposed architecture is for the PMOS transistor M_P1_. As from the circuitry, the gate terminal of the NMOS transistor M_N1_ is connected to the output of the rectifier to obtain the threshold compensation. *C_P_* is a pumping capacitor while C_L_ is used as a battery for charge storing purposes, while R_L_ is the load resistor. Two NMOS transistors (M_N2_ and M_N3_) and one PMOS (M_P2_) are auxiliary transistors operating in the subthreshold region. These transistors provide an optimum gate to source compensation voltage to the main transistor M_P1_. To produce this optimum gate to source compensation voltage, proper sizing of the auxiliary transistors is needed, while the leakage current can be minimized by a high impedance path to the ground. In practice, to obtain high output voltage, a transistor with very small resistance is needed. Therefore, the optimization should be carried out under the worst condition for RF energy harvesting applications. The leakage and parasitic loss increase as the size of the transistor increases. Similarly, by the selection of small transistors, an improper and undesirable transfer of charges occur. For this purpose, the width of the transistors M_N1_ and M_P1_ are chosen as 8 and 16 µm, respectively, while their channel lengths are chosen as a minimum. The sizes of the auxiliary transistors M_P2_, M_N2_, and M_N3_ are chosen as 1 µm/8 µm, 1 µm/4 µm, and 1 µm/2 µm, respectively. The capacitors’ values of *C_P_* and C_L_ are set as 400 fF and 1 pF. The *PCE* has been decreasing by further increasing the sizes of the transistors. The sizes of the transistors are selected to achieve high efficiency with a low input power range.

**Figure 4:**
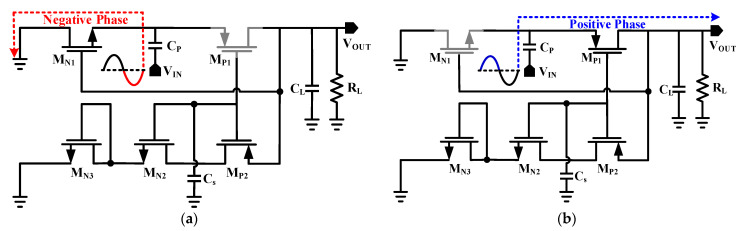
Operating principle of the RF-DC converter. (**a**) Charging (negative) phase. (**b**) Discharging (positive) phase.

Figure 4 shows the operating principle of the proposed RF-DC converter. The RF-DC converter enters the charging phase (Figure 4a), as the negative input signal appears. In the negative phase, the NMOS transistor *M_N_*_1_ will first go into conducting mode, and then the capacitor *C_P_* will begin to charge. The voltage produced across *C_P_* can be found by applying Kirchhoff’s voltage law (KVL) as:

(4) $$V_{CP}=-V_{IN}+V_{dMN1}$$

where *V_IN_* is the peak amplitude of the input RF voltage and *V_dN_* is the voltage drop across the transistor *M_N_*_1_ due to the threshold voltage. By considering *C_P_* as an ideal capacitor, the whole charge will be transferred to the C_L_ without any loss during the discharging phase. The first-order equivalent circuit of the second path during the discharging phase of *C_P_* is depicted in Figure 4b to determine the voltage across *C_AUX_*. Thus, by applying KVL, we can write:

(5) $$V_{OUT}=V_{AUX}-V_{th}+I_{d}R_{ON}-V_{IN}$$

where *V_AUX_* is a voltage that appears across the auxiliary capacitor *C_AUX_*, *V_th_* is the threshold voltage, and the *R_ON_* is the ON resistance of the transistor M_P1_ while the drain current is represented by *I_d_*. The *I_d_* is also given by the equation:

(6) $$I_{d}=\beta {\left(V_{AUX}-V_{th}\right)}^{2}$$

Principally, “*β*” is equal to (µ_n_*C*_0_/2*L*^2^) where *µ_n_* is the mobility of the electron in the channel, the capacitance between the channel and gate of the transistor is *C*_0_, and *L* is the length of the source to the drain channel. Several different properties affect the threshold voltage of the MOS structure. The presence of a threshold voltage, for example, can be a major constraint in circuits built to work with low-voltage batteries. As a result, substantial effort was directed toward developing MOS architectures with low *V_th_* values. By solving Equation (4) for V_CL_, we get

(7) $$V_{AUX}=V_{th}-\frac{1}{\beta R}+\sqrt{{\left(\frac{1}{\beta R}\right)}^{2}+4\left(V_{OUT}+V_{IN}\right)}$$

Equation (7) shows that the voltage across *C_AUX_* and *V_AUX_* is developed using the contribution of both input and output voltages. The rectifier will enter the discharging phase as the positive RF signal appears. Figure 4b shows the equivalent circuit for the discharging phase of the rectifier. It was shown clearly in V_CL_ that the voltage is the DC biasing gate for the source voltage of the transistor M_N1_. During this phase, the threshold voltage compensation in transistor M_P1_ occurs. Applying KVL on this discharging path and replacing Equation (3) will result as:

(8) $$V_{OUT}=2V_{IN}-V_{dMN1}-V_{dMP1}$$

where *V_dMP_*_1_ is ideally compensated by *V_AUX_*, hence, we can also write Equation (7) as

(9) $$V_{OUT}=2V_{IN}-V_{dMN1}$$

As a result, it can be deduced that, ideally, *M_N_*_1_ will account for the majority of the loss in the rectified output DC voltage obtained by the proposed RF-DC converter.

## 4. Measurement Results

**Figure 5:**
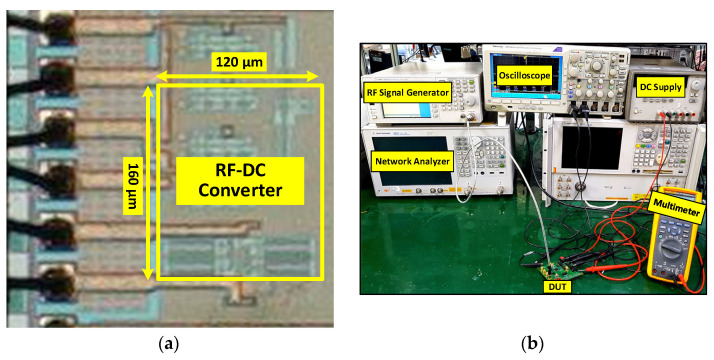
(**a**) Chip microphotograph and (**b**) measurement setup of the proposed RF-DC converter architecture.

The proposed self-threshold voltage cancellation RF-DC converter is designed and implemented in standard 180 nm CMOS technology. Figure 5a shows the fabricated chip micrograph. The total active area of the fabricated chip is 160 × 120 µm excluding the pads. The fabricated chip is soldered and packaged on an FR4 PCB board. Figure 5b shows the measurement setup and the fabricated chip is measured with a single-tone sinusoidal signal of 900 MHz and 2.4 GHz generated by the signal generator (Agilent E4438C). The output DC voltage is measured using an oscilloscope and digital multi-meter. The impedance matching network is integrated off-chip between the 50 Ω signal generator and the fabricated chip. The impedance matching network enhances the incoming RF signal and transfers it to the chip. Reflection between the impedance matching circuit and the RF-DC converter, PCB trace losses, and impedance matching network losses caused by passive elements, are the factors that affect the overall performance of the RF-DC converter. The net input power can be calculated after excluding all these losses and transfers to the chip. The maximum output DC voltage and the *PCE* were measured for the resistive loads of 100 kΩ, 500 kΩ, and 1 MΩ.

**Figure 6:**
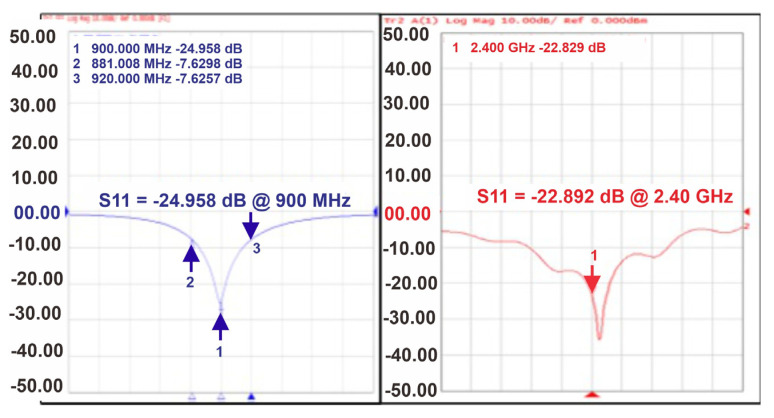
Measured |*S*11| for the RF-DC converter at a 1 MΩ load.

Figure 6 shows the measured |S11| parameter for the proposed RF-DC converter. The measured values of |S11| at 900 MHz and 2.4 GHz are −24.958 and −22.892 dB, respectively, for the 1 MΩ load which shows excellent matching.

**Figure 7:**
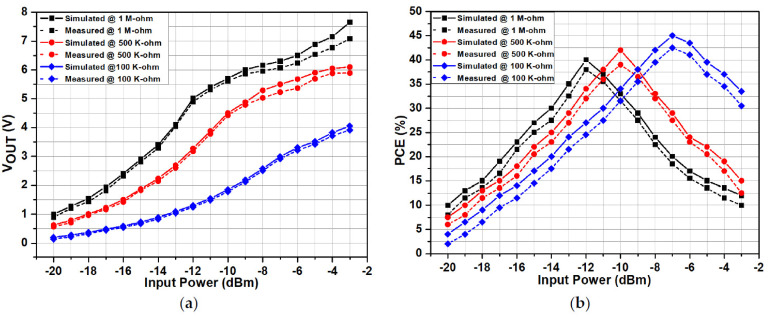
Simulated and measured results of the RF-DC converter for different loads. (**a**) Output DC voltage and (**b**) *PCE*.

The performance of the proposed RF-DC converter can be measured by the output DC voltage and its efficiency. It achieves better efficiency and output voltage with very low input power. The input power is varied from −20 to −3 dBm. The output rectified DC voltage after simulation and measurement results are plotted across various resistive loads, as shown in Figure 7a. By increasing the load resistance, the output DC voltage of the proposed RF-DC circuit increases gradually. Similarly, Figure 7b shows the simulated and measured power conversion efficiency of the proposed RF-DC converter as a function of input power for different load resistances for the 900 MHz frequency. The proposed architecture achieves maximum simulated efficiency of 45% and measured efficiency of 42.5% with −7 dBm input power level and 100 KΩ of load resistance. However, the *PCE* starts decreasing by further increasing the input power level. For a load of 500 KΩ, the maximum simulated efficiency is 42.5% and the measured efficiency is 39% at −10 dBm of input power level.

Similarly, a simulated efficiency of 40% and measured efficiency of 38% is achieved at −12 dBm of the input power level at 1 MΩ load resistance. If the load resistance increases, the efficiency curve shifts towards the left side.

**Figure 8:**
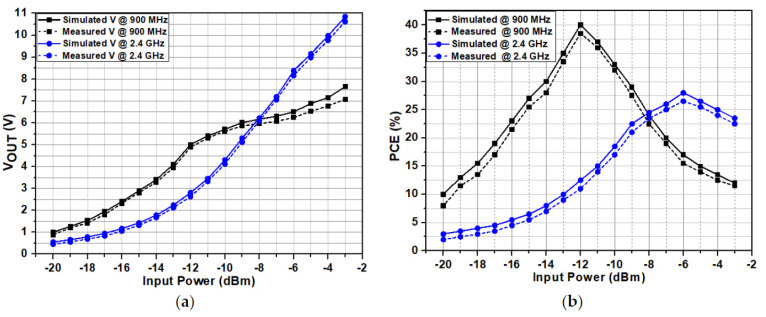
Simulated and measured results of the RF-DC converter at different frequencies for 1 MΩ. (**a**) Output DC voltage and (**b**) *PCE*.

**Figure 9:**
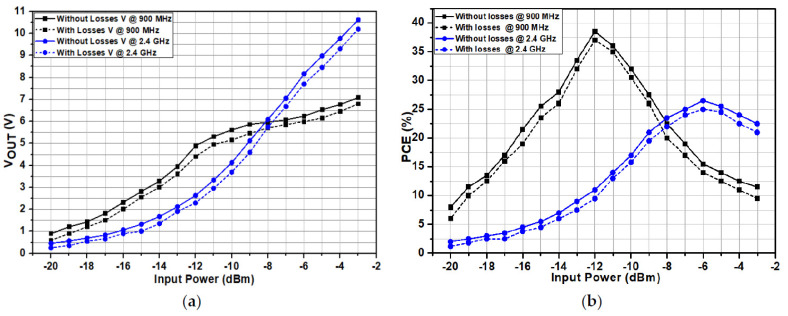
Measured results of the RF-DC converter at different frequencies for 1 MΩ with and without losses. (**a**) Output DC voltage and (**b**) *PCE*.

The performance of the proposed RF-DC converter was checked in all PVT variations (typical corner points, slow corner points, and fast corner points) and compared with the measurement results. The performance was first checked on simulation and post-simulation levels for PVT variations and then measurement level. The proposed RF-DC converter satisfied both the simulation and measurement results. Figure 8 shows simulated and measured results of the RF-DC converter at different frequencies, i.e., 900 MHz and 2.4 GHz. Figure 8a presents the simulated and measured DC voltage at both 900 MHz and 2.4 GHz frequencies. The recorded simulated and measured output DC voltage is 5 and 4.8 V, respectively, at −12 dBm input power level for 900 MHz frequency. An 8.38 V simulated and 8.1 V measured voltage is achieved for 2.4 GHz frequency at −6 dBm input power level. Similarly, for 2.4 GHz, the maximum simulated and measured efficiencies are 28% and 26.5% at −6 dBm input power level, as shown in Figure 8b. Moreover, the proposed architecture obtains high *PCE* for a wide input power range. The sensitivity of the proposed architecture to obtain a 1 V output DC voltage is −20 dBm for 900 MHz. Figure 9 shows the measured results including the output DC voltage and *PCE* of the proposed RF-DC converter including all losses for different frequencies with 1 MΩ load resistance for both 900 MHz and 2.4 GHz frequencies. Figure 9a shows the output measured voltage at both 900 MHz and 2.4 GHz frequencies.

The measured voltage including a loss at 900 MHz frequency is 4.24 V with a −12 dBm input power level. Similarly, a 7.3 V is achieved at a 2.4 GHz frequency with a −6 dBm input power level including all losses, respectively. As the output voltage is relatively high, therefore, the breakdown voltage of the transistors (MN1 and MP1) used in the main rectification chain is large enough to bear the voltage larger than 5 V. Figure 9b shows the *PCE* of the proposed RF-DC converter including all the losses at both 900 MHz and 2.4 GHz frequencies. The maximum *PCE* achieved at 900 MHz including losses is 37%. Similarly, a 25% efficiency is achieved at 2.4 GHz frequency including all the losses.

Table 1 shows the summary of the performance of the proposed architecture and compares it with the prior work. The proposed circuit shows decent performance and minimum active area. At −12 and −6 dBm input powers, the proposed RF-DC converter achieves maximum efficiency of 38.5% and 26% for the 1 MΩ load resistance. Moreover, the proposed RF-DC converter maintains more than 20% *PCE* from the −8 to −16 dBm input power range. It can be seen that the DC output voltage achieved through our work, i.e., 4.8 V for 900 MHz and 8.1 for V for 2.4 GHz is higher than the compared reference works. However, the circuit reported in [26] shows higher sensitivity than this work but the achieved *PCE* is much lower than our proposed architecture. Overall, our proposed circuit shows better performance and sensitivity than the previous architectures.

## 5. Conclusions

This paper presented a self-threshold voltage (*V_th_*) compensated RF-DC converter operating at 900 MHz and 2.4 GHz frequencies for RF energy harvesting applications. The proposed RF-DC converter was implemented in 180 nm CMOS technology. In the proposed RF-DC circuit, the auxiliary transistors and output DC voltage generated the bias voltage for the *V_th_* compensation of the rectifying devices in the main rectification chain. The measurement results showed that the proposed circuit obtained a peak *PCE* of 38.5% at −12 dBm input power across a 1 MΩ load for 900 MHz frequency. Similarly, for 2.4 GHz, the proposed RF energy harvester showed a peak *PCE* of 26.5% at −6 dBm across a 1 MΩ load resistance.

## Figures and Tables

**Table 1:** Summary and performance comparison.

Parameters	This Work	[2]	[13]	[18]	[22]	[23]	[24]
		2015	2019	2017	2017	2020	2014
**Technology (nm)**	**180**	130	Diode-Based	65	180	180	130
**Energy harvesting**	**RF**	RF	RF	RF	RF	RF	RF
**Frequency (GHz)**	**0.9/2.4**	0.902~0.928	2.45	0.953	0.915	0.902	0.915
**Load (MΩ)**	**1**	1	0.2	0.147	1	0.2	1
**Input power (dBm)**	**−12/−6**	−15	13	−10	−2	−8	−16.8
**DC output (V)**	**4.8/8.1**	3.2	1.7	2.6	2.4	3.23	2.2
***PCE* (%)**	**38.5/26.5**	32	37.5	36.5	27	33	22.6
**Effective Area**	**0.19 mm^2^**	-	0.74 mm^2^	0.47 mm^2^	-	0.105 mm^2^	-
**Sensitivity: 1 V for 1 MΩ**	**−20 dBm**	−20.5 dBm	-	−17.5 dBm	14.8 dBm	−20.2 dBm	−21.6 dBm

## Data Availability

Not applicable.
